# Feeding ecology of *Ellochelon vaigiensis* (Quoy & Gaimard, 1825) living in the Mekong Delta, Vietnam

**DOI:** 10.1002/ece3.9352

**Published:** 2022-09-20

**Authors:** Quang Minh Dinh, Ngon Trong Truong, Ton Huu Duc Nguyen, Tran Thi Huyen Lam, Tien Thi Kieu Nguyen, Dung Quang Le, Simon Kumar Das

**Affiliations:** ^1^ Department of Biology, School of Education Can Tho University Can Tho Vietnam; ^2^ Department of Molecular Biotechnology, Biotechnology Research and Development Institute Can Tho University Can Tho Vietnam; ^3^ Institute of High Quality Biotechnology‐Food Technology Cuu Long University Vinh Long Vietnam; ^4^ Department of Biology An Khanh High School Can Tho Vietnam; ^5^ Institute for Circular Economy Development Vietnam National University ‐ Ho Chi Minh City Thu Duc City Vietnam; ^6^ Department of Earth Sciences and Environment, Faculty of Science and Technology Universiti Kebangsaan Malaysia Bangi Malaysia

**Keywords:** algi‐omnivorous, diet composition, feeding biology, feeding intensity, squaretail mullet

## Abstract

*Ellochelon vaigiensis* (squaretail mullet) adapts to a wide salt spectrum, grows quickly and is easy to raise with other species, so it is the object of attention in aquaculture. Information on the biology and ecology of the species, diet, in particular, is still scattered. Here, we explore the feeding habit, feeding intensity, and food composition of the squaretail mullet. A total of 942 fish (526 males and 416 females) were collected from November 2020 to October 2021 at four coastal sites in the Mekong Delta, Vietnam. The squaretail mullet is an algi‐omnivorous fish, as their relative gut length (1.81) falls into the range 1–3, and the diet is mostly based on algae. The feeding intensity is high due to the high value of the fullness index (4.39 ± 0.08%). The fullness index did not vary by sampling site and month, while RGL and FI changed depending on sex. Bacillariophyta (49.13%), detritus derived from organic matter (30.37%), and Cyanophyta (18.39%) are the dominant food items in the diet composition of *E. vaigiensis*, in which detritus is the most important food with the highest IRI index. Besides, Euglenophyta (1.00%), Chlorophyta (0.95%), Paramecium (0.06%), Copepoda (0.04%), Rotatoria (0.03%), Polychaeta (0.02%), and Cladocera (0.01%) are also recorded and ranked based on their biovolume. Some differences in diet composition are observed between immature and mature at different seasons and their interactions. Our results increase the knowledge about the feeding ecology of squaretail mullet and can help the sustainable management of this commercially important fish species.

## Graphical abstract

The results denote that squaretail mullet is an algi‐omnivorous fish, as their relative gut length (1.81) falls into the range 1‐3, and the diet is mostly based on algae. The feeding intensity of this mullet is high due to the high value of the fullness index (4.39 ± 0.08%). The results obtained from this study increase the knowledge about the feeding ecology of squaretail mullet and can help the sustainable management of this commercially important fish species in the MD.

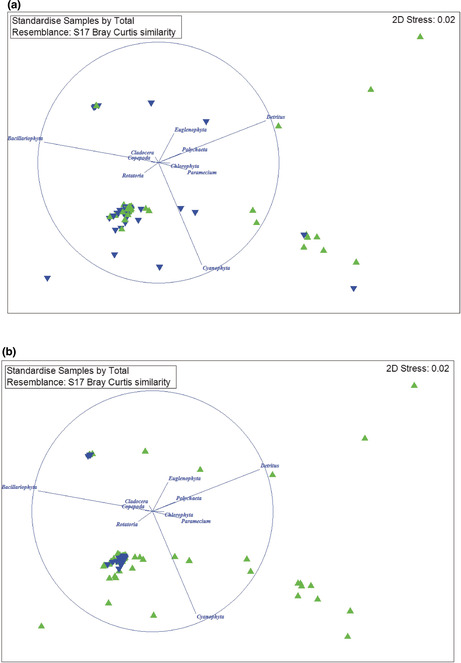

## 1 INTRODUCTION

The square mullet [*Ellochelon vaigiensis* (Quoy & Gaimard, 1825)] is an oily fish with few bones (Brian, 2017). However, their value products are not fish fleshes but bait fisheries and the roe‐carrying females (Ben Khemis et al., 2019). Dried and salted roe has become a high‐value product with increasing demand globally (Bledsoe et al., 2003). *Ellochelon vaigiensis* is a suitable candidate for brackish aquaculture because its growth rate, maximum length, and condition factor are all high.

Grant and Spain (1975) show that *E. vaigiensis's* spawning season is February to March in Northern Queensland, Australia, with batch fecundity of 0.805–1.204 × l0^6^ and oocyte diameter of 0.54 ± 0.05 mm, whereas in Krusadai Island, Gulf of Mannar, the spawning season lasts from May to February (Chidambaram & Kuriyan, 1952). Brian (2017) reported the gut length of this mullet is several times larger than the body length of the squaretail mullet. In Negombo Lagoon on the west coast of Sri Lanka, the relative gut length value of this species ranges from 2.31 to 2.43, and the food source of this fish is quite diverse, including diatoms, green algae, blue‐green algae, dinoflagellates, sand grains, detritus, mollusks, crustaceans and polychaetes (Wijeyaratne & Costa, 1990). Its digestive tract is described by (Anh et al., 2022), and fish sex can be differentiated using the relationship between body high and head length (Linh et al., 2022).

Research on food composition and feeding habits of fish is helpful to evaluate better fish stocks, ecosystem patterns, and nutritional relationships in aquatic communities (Mohamed, 2014). Food and feeding habits change throughout the day, season, body size, habitat, and food sources available (Fatema et al., 2013). In addition, relative gut length (RGL) can help determine whether a fish is herbivorous, carnivorous, omnivorous, herbi‐omnivorous, or carni‐omnivorous (Al‐Hussaini, 1947). The fullness index (FI) examines the feeding intensity of fish (Desai, 1970) and helps to understand the interrelationships between fish and ecosystems as well as the impact of the environment on fish feeding patterns (Sajeevan & Kurup, 2013). Hence, we analyze the food composition, feeding habits, and intensity of squaretail mullet in the Mekong Delta, which could be helpful as a supplement to the existing information on the nutritional biology of *Ellochelon vaigiensis*.

## 2 MATERIALS AND METHODS

### 2.1 Fish collection and analysis

**FIGURE 1:**
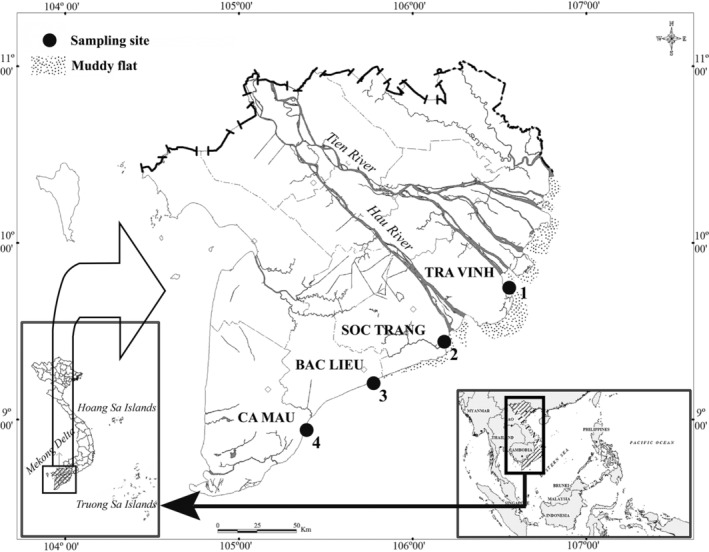
Map of the Mekong Delta showing the sampling sites (•: Sampling area; 1: Thanh Phu, Ben Tre; 2: Duyen Hai, Tra Vinh; 3: Tran De, Soc Trang; 4: Dong Hai, Bac Lieu; modified from Dinh (2018)).

From November 2020 to October 2021, fish samples were collected monthly (by trawl net with a length of 10 m, a height of 2 m, and mesh size of 2.0 cm) at four sites in Mekong Delta, including Thanh Phu, Ben Tre (9°57′01.3″N 106°31′43.1″E); Duyen Hai, Tra Vinh (9°40′29.5″N 106°34′49.5″E); Tran De, Soc Trang (9°26′19.7″N 105°10′48.1″E); and Dong Hai, Bac Lieu (9°05′50.5″N 105°29′54.7″E) (Figure 1). Four sampling sites were in the lower reaches of the Hau river adjacent to the East Sea where mullet species have been found. The typical tidal regime in the sampling areas is semi‐tidal, and the mean annual temperature is about 27°C (24–33°C). There are only two seasons: the wet season lasting from June to December and the dry season lasting from January to May. About 90% is concentrated in the wet season, and the remaining 10% is scattered in the dry season (Le et al., 2006).

We sampled 30 fish per month with a minimum length of 15 cm. Specimens were anesthetized with MS222 and immediately kept in ice before transporting to the laboratory. Specimens were identified to species based on the caudal fin and external morphology (Tran et al., 2013) before measuring total length, body weight, digestive tract length and weight. The relative gut length (RGL) was calculated as the ratio of gut length/fish total length. The feeding intensity was determined from the fullness index, computed as FI = 100 × W_i_/W, where Wi is gut weight, and W is body weight (Herbold, 1986).

The taxonomic composition of the stomach contents was determined under the microscope, according to Nguyen et al. (2013). For each prey category, the frequency of prey occurrence was calculated as %O_i_ = 100 × Oi/N (O_i_ is the number of fish consuming prey i and N is the total number of fish examined) (Hynes, 1950). Moreover, the percentage in number for each prey was computed: %N_i_ = 100 × N_i_/N_total_ (N_i_ is the number of prey i, N_total_ is the number of all prey individuals) (Hyslop, 1980). The method of point of prey or biovolume was calculated according to the formula %Vi = (100 × O_i_ × N_i_)/(∑O_i_ × N_i_) (V_i_, O_i,_ and N_i_ are the percentage of biovolume, occurrence, and the number of prey i, respectively) (Natarajan & Jhingran, 1961).

To determine the importance of types of food in the diet composition, we calculated the percentage of the important relative index (%IRI) of each food item as follows: %IRI = %O × (%N + %V) (IRI, O, N, and V are the percentage of the index of relative importance, occurrence, occurrence, and biovolume of prey, respectively) (Windell, 1971).

### 2.2 Data analysis

The normal distribution of RGL and FI was verified by Kolmogorov–Smirnov test (Kim, 2015). Since the data were not normally distributed, the effects of sex, body size, season, site, and their interactions on the variation of RGL and FI were qualified by the generalized linear model (GLM) with the Wald chi‐square test.

Fish length at first maturity (*L*_*m*_) was used to divide fish into the immature group (*TL* < *L*_*m*_) and the mature group (*TL* ≥ *L*_*m*_). The male and female *L*_*m*_ was calculated as P = 1/(1 + exp[−r × (*TL*‐*Lm*)]) (P: proportion of mature individuals in a length class; *TL*: fish total length; and r: model parameter) (Zar, 1999).

Dietary changes by sex, body size, season, and site based on biovolume percentage of foods were analyzed in the software PRIMER v.6.1.11 with PERMANOVA+v.1.0.1, according to Dinh et al. (2017). After inputting into PRIMER, the data were standardized using the tool “Standardise Samples by Total” and constructed a resemblance matrix using the tool “S17 Bray Curtis similarity.” At the same time, the Monte Carlo test was applied when analyzing PERMANOVA to limit errors in the analysis process. The MDS (Multidimensional Scaling) analysis was used to visualize the prey's contribution to diet composition. Since the diet composition changed with sex, body size, and season, the Mann–Whitney U test was used to confirm which prey contributed to the differences. When the diet composition changed with site, the Kruskal–Wallis H test was used to verify which prey contributed to the differences. All tests were performed at the 95% confidence level.

## 3 RESULTS

### 3.1 Relative gut length (RGL)

Four hundred sixteen females and 526 males out of 942 squaretail mullets were used to analyze the correlation between the alimentary tract and fish total length. The distribution of RGL values was not normal (Kolmogorov–Smirnov test, *KS* = 0.04, *p* = .004). The overall relative gut length (RGL) was 1.81 ± 0.01, ranging from 1 to 3; therefore, the species was an omnivore (Al‐Hussaini, 1947).

The *L*_*m*_ values of males and females in Thanh Phu, Ben Tre were 10.6 cm and 16.7 cm; in Duyen Hai, Tra Vinh were 17.7 cm, 19.3 cm; in Tran De, Soc Trang were 13.9 cm, 13.8 cm; and in Dong Hai, Bac Lieu were 16.1 cm, 17.7 cm (unpublished data). The RGL exhibited significant differences according to sex but not by body size, season, and sites. Specifically, female RGL (1.89 ± 0.03) was significantly higher than that of males (1.73 ± 0.03) (GLM, *W*_*t*_ = 17.44, *df* = 1, *p* < .01). Immature and mature fishes exhibited RGL values not statistically different (1.80 ± 0.03 and 1.81 ± 0.02, respectively, *W*_*t*_ = 0.02, *df* = 1, *p* = .90). This can suggest that the feeding habit did not change from juvenile to adult. The feeding pattern of the squaretail mullet in the dry season (1.81 ± 0.03) was not significantly different from that of the wet season (1.80 ± 0.02) (*W*_*t*_ = 0.19, *df* = 1, *p* = .67). In addition, squaretail mullets did not change their feeding habits at the four sampling sites as their RGL values ranged from 1 to 3 (RGL in Ben Tre, Tra Vinh, Soc Trang, and Bac Lieu were 1.79 ± 0.05, 1.75 ± 0.03, 1.83 ± 0.04, and 1.85 ± 0.04, respectively) (*W*_*t*_ = 5.26, *df* = 3, *p* = .15). The RGL was independent on the interactions of sex × body size (*W*_*t*_ = 1.01, *df* = 1, *p* = .32), sex × season (*W*_*t*_ = 0.01, *df* = 1, *p* = .93), sex × site (*W*_*t*_ = 3.57, *df* = 3, *p* = .31), body size × season (*W*_*t*_ = 1.71, *df* = 1, *p* = .19), body size × site (*W*_*t*_ = 4.32, *df* = 3, *p* = .23), season × site (*W*_*t*_ = 2.36, *df* = 3, *p* = .50).

### 3.2 The fullness index (FI)

The distribution of FI values was not normal (*KS* = 0.106, *p* < .01). This value of the squaretail mullet was relatively high, about 4.39 ± 0.08%, indicating that the mullet has a high feeding intensity. The differences in body size (GLM, *W*_*t*_ = 3.58, *df* = 1, *p* = .06), season (*W*_*t*_ = 0.01, *df* = 1, *p* = .92), and sites (*W*_*t*_ = 5.81, *df* = 3, *p* = .12) did not affect FI values. The FI of females (4.20 ± 0.12%) was statistically lower than that of males (4.55 ± 0.12%) (*W*_*t*_ = 4.12, *df* = 1, *p* = .04), whereas the FI of immature (4.53 ± 0.22%) was equivalent to mature (4.04 ± 0.14%). Similarly, this value did not differ between the dry season (4.26 ± 0.21%) and the wet season (4.29 ± 0.14%). The analysis results also showed that FI fluctuated not significantly amongst Ben Tre (4.71 ± 0.39%), Tra Vinh (4.27 ± 0.17%), Soc Trang (3.84 ± 0.20%), and Bac Lieu (4.32 ± 0.25%). The interactions of site × season (*W*_*t*_ = 16.33, *df* = 3, *p* = .001) and sex × body size (*W*_*t*_ = 6.22, *df* = 1, *p* = .01) effected on FI. The remaining interactions showed no significant effect on FI such as site × sex (*W*_*t*_ = 1.46, *df* = 3, *p* = .69), site × body size (*W*_*t*_ = 1.79, *df* = 3, *p* = .62), sex × season (*W*_*t*_ = 0.53, *df* = 1, *p* = .47), and season × body size (*W*_*t*_ = 2.97, *df* = 1, *p* = .09).

### 3.3 General diet composition

The food items were classified into 10 categories: Bacillariophyta, detritus derived from organic matter, Cyanophyta, Euglenophyta, Chlorophyta, *Paramecium*, Copepoda, Rotatoria, Polychaeta, and Cladocera (Table 1). The frequency of Bacillariophyta, detritus derived from organic matter, Cyanophyta, Euglenophyta, and Chlorophyta were not much different, accounting for a significantly higher percentage than the rest of the food items. Notably, the biovolume analysis showed that Bacillariophyta accounted for the highest, up to 49.13%, followed by detritus derived from organic matter (30.37%) and Cyanophyta (18.39%). Although the occurrence in the digestive tract of squaretail mullet was with high frequency, biovolume of Euglenophyta and Chlorophyta were relatively low.

**TABLE 1:** Frequency of occurrence (O), number (N), biovolume (V), and important relative index (IRI) (%) of food items in squaretail mullet

Category		Detritus	Bacillariophyta	Cyanophyta	Chlorophyta	Euglenophyta	*Paramecium*	Polychaeta	Cladocera	Copepoda	Rotatoria
%O	Male	18.98	18.98	18.90	18.47	18.18	1.73	0.90	0.54	1.52	1.80
	Female	18.93	18.93	18.75	18.30	18.07	1.91	0.59	1.05	1.37	2.09
%N	Male	53.57	28.77	16.16	0.85	0.60	0.02	0.01	0.00	0.01	0.01
	Female	53.41	28.91	16.22	0.83	0.58	0.02	0.00	0.00	0.01	0.01
%V	Male	30.44	49.04	18.36	0.96	1.02	0.07	0.03	0.01	0.04	0.03
	Female	30.31	49.21	18.41	0.95	0.99	0.06	0.01	0.01	0.03	0.03
%IRI	Male	42.10	39.00	17.24	0.88	0.77	0.00	0.00	0.00	0.00	0.00
	Female	41.99	39.18	17.21	0.86	0.75	0.00	0.00	0.00	0.00	0.00
%O	Immature	18.85	18.85	18.64	18.43	18.07	1.61	0.67	0.93	1.92	2.02
	Mature	19.03	19.03	18.96	18.37	18.17	1.94	0.82	0.66	1.15	1.87
%N	Immature	53.43	28.84	16.22	0.84	0.59	0.04	0.00	0.00	0.02	0.02
	Mature	53.51	28.84	16.18	0.84	0.59	0.02	0.00	0.00	0.01	0.01
%V	Immature	30.31	49.07	18.40	0.96	1.00	0.10	0.02	0.01	0.07	0.05
	Mature	30.39	49.14	18.38	0.95	1.00	0.05	0.02	0.01	0.03	0.03
%IRI	Immature	42.03	39.11	17.19	0.89	0.76	0.01	0.00	0.00	0.00	0.00
	Mature	42.04	39.07	17.26	0.87	0.76	0.00	0.00	0.00	0.00	0.00
%O	Dry season	18.97	18.97	18.82	18.25	17.99	1.56	1.19	0.57	1.61	2.07
	Wet season	18.95	18.95	18.85	18.49	18.22	1.97	0.49	0.89	1.35	1.84
%N	Dry season	53.83	28.52	16.25	0.84	0.51	0.03	0.00	0.00	0.01	0.01
	Wet season	53.32	29.01	16.16	0.84	0.63	0.02	0.00	0.00	0.01	0.01
%V	Dry season	30.69	48.78	18.53	0.96	0.87	0.07	0.02	0.01	0.04	0.03
	Wet season	30.21	49.31	18.31	0.95	1.07	0.06	0.02	0.01	0.04	0.03
%IRI	Dry season	42.39	38.77	17.30	0.87	0.66	0.00	0.00	0.00	0.00	0.00
	Wet season	41.86	39.25	17.19	0.88	0.82	0.00	0.00	0.00	0.00	0.00
%O	Thanh Phu, Ben Tre	19.02	19.02	18.84	18.13	18.04	1.79	0.98	0.71	1.52	1.96
	Duyen Hai, Tra Vinh	18.92	18.92	18.92	18.27	18.06	1.64	0.93	0.93	1.43	2.00
	Tran De, Soc Trang	18.98	18.98	18.80	18.71	18.17	2.09	0.54	0.73	1.18	1.82
	Dong Hai, Bac Lieu	18.93	18.93	18.78	18.49	18.26	1.78	0.59	0.67	1.63	1.93
%N	Thanh Phu, Ben Tre	54.08	28.22	16.20	0.83	0.61	0.03	0.00	0.00	0.01	0.01
	Duyen Hai, Tra Vinh	53.37	28.96	16.27	0.83	0.52	0.02	0.00	0.00	0.01	0.01
	Tran De, Soc Trang	53.42	29.06	15.99	0.86	0.62	0.03	0.00	0.00	0.01	0.01
	Dong Hai, Bac Lieu	53.26	28.98	16.24	0.84	0.63	0.02	0.00	0.00	0.01	0.01
%V	Thanh Phu, Ben Tre	30.91	48.39	18.51	0.95	1.05	0.07	0.02	0.01	0.04	0.03
	Duyen Hai, Tra Vinh	30.28	49.29	18.46	0.95	0.88	0.05	0.02	0.01	0.03	0.03
	Tran De, Soc Trang	30.27	49.40	18.12	0.97	1.06	0.08	0.01	0.01	0.04	0.04
	Dong Hai, Bac Lieu	30.17	49.24	18.40	0.95	1.08	0.06	0.02	0.01	0.04	0.03
%IRI	Thanh Phu, Ben Tre	42.65	38.44	17.25	0.86	0.79	0.00	0.00	0.00	0.00	0.00
	Duyen Hai, Tra Vinh	41.89	39.18	17.39	0.86	0.67	0.00	0.00	0.00	0.00	0.00
	Tran De, Soc Trang	41.98	39.36	16.95	0.90	0.81	0.01	0.00	0.00	0.00	0.00
	Dong Hai, Bac Lieu	41.83	39.22	17.24	0.87	0.83	0.00	0.00	0.00	0.00	0.00

The species *E. vaigiensis* was a herbi‐omnivorous fish, ingesting mostly detritus derived from organic matter, Bacillariophyta, and Cyanophyta based on their %IRI (42.04, 39.09, and 17.23, respectively) (Table 1). Two preys, including Euglenophyta and Chlorophyta, were considered as less important preys in small numbers. Meanwhile, Paramecium, Copepoda, Rotatoria, Polychaeta, and Cladocera were non‐essential prey.

### 3.4 Variation in diet composition according to sex, fish size, season, and sampling sites

**FIGURE 2:**
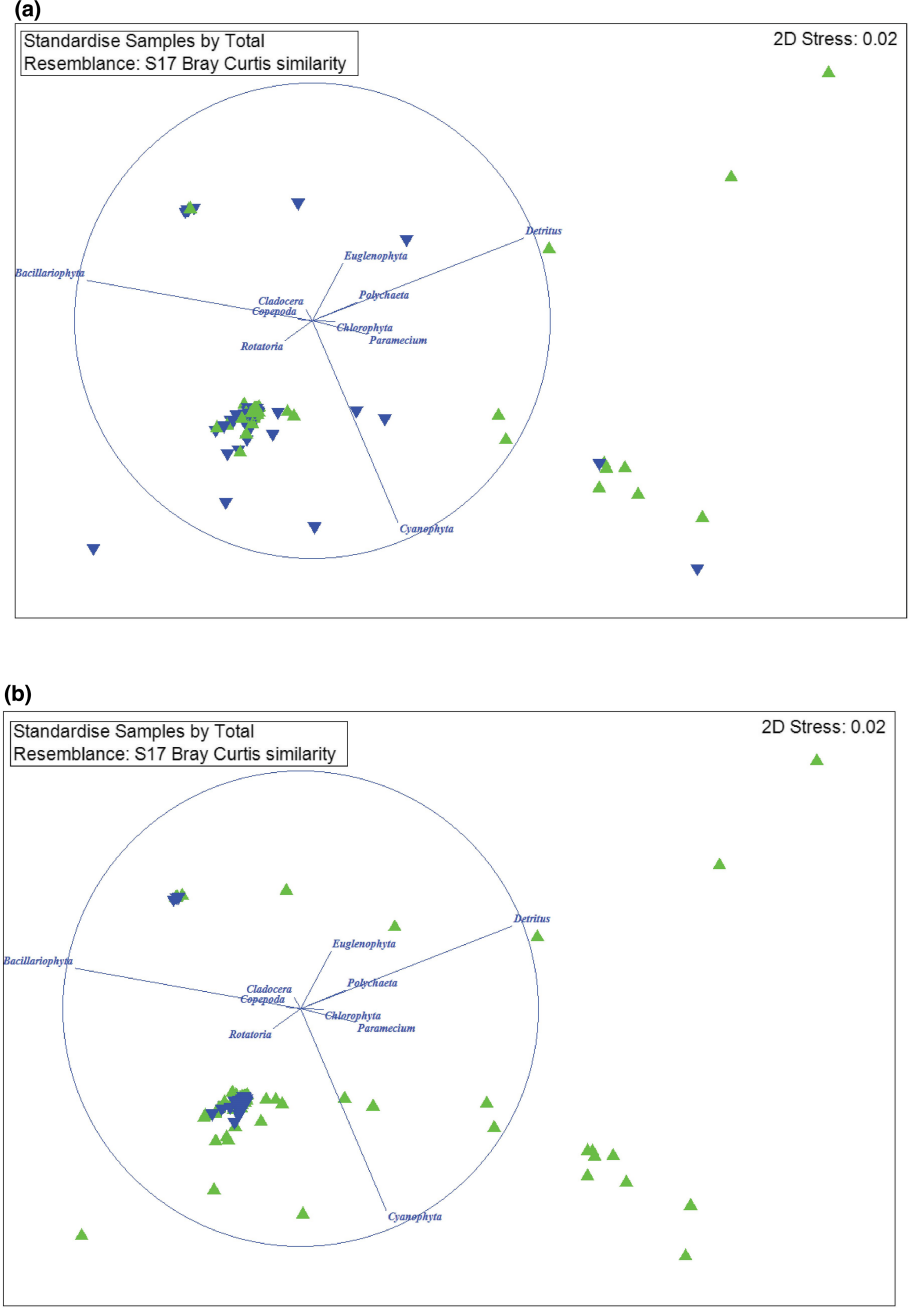
Multidimensional Scaling (MDS) analysis of diet composition of squaretail mullet according to body size (a) (green triangle: mature fish; blue triangle: immature fish) and season (b) (green triangle: dry season; blue triangle: wet season).

Fish size (PERMANOVA, *Pseudo‐F* = 2.61, *df* = 1, *p* = .05) and sampling season (*Pseudo‐F* = 19.42, *df* = 1, *p* = .001) significantly affected diet composition. The result of MDS visualized the food composition according to body size and seasons and revealed that Bacillariophyta, detritus derived from organic matter, and Cyanophyta were the three main foods (Figure 2a and b, respectively). MDS graphs were reliable because the 2D stress values in Figure 2 were 0.02.

The result of the Mann–Whitney test revealed that immature fish ingested more Copepoda (0.12 ± 0.03%) than mature fish (0.05 ± 0.01%) (Mann–Whitney U, *Z* = −2.42, *df* = 1, *p* = .02), whereas they ate other foods equally. In dry season, this mullet consumed more detritus (*Z* = −7.18, *df* = 1, *p* < .01), Cyanophyta (*Z* = −5.87, *df* = 1, *p* < .01), Chlorophyta (*Z* = −2.17, *df* = 1, *p* = .03), and Polychaeta (*Z* = −2.79, *df* = 1, *p* = .005) than in wet season. Otherwise, the squaretail mullet ate more Bacillariophyta (*Z* = −3.86, *df* = 1, *p* < .01) and Euglenophyta (*Z* = −7.42, *df* = 1, *p* < .01). No differences were found in the other prey items between dry and wet seasons.

**FIGURE 3:**
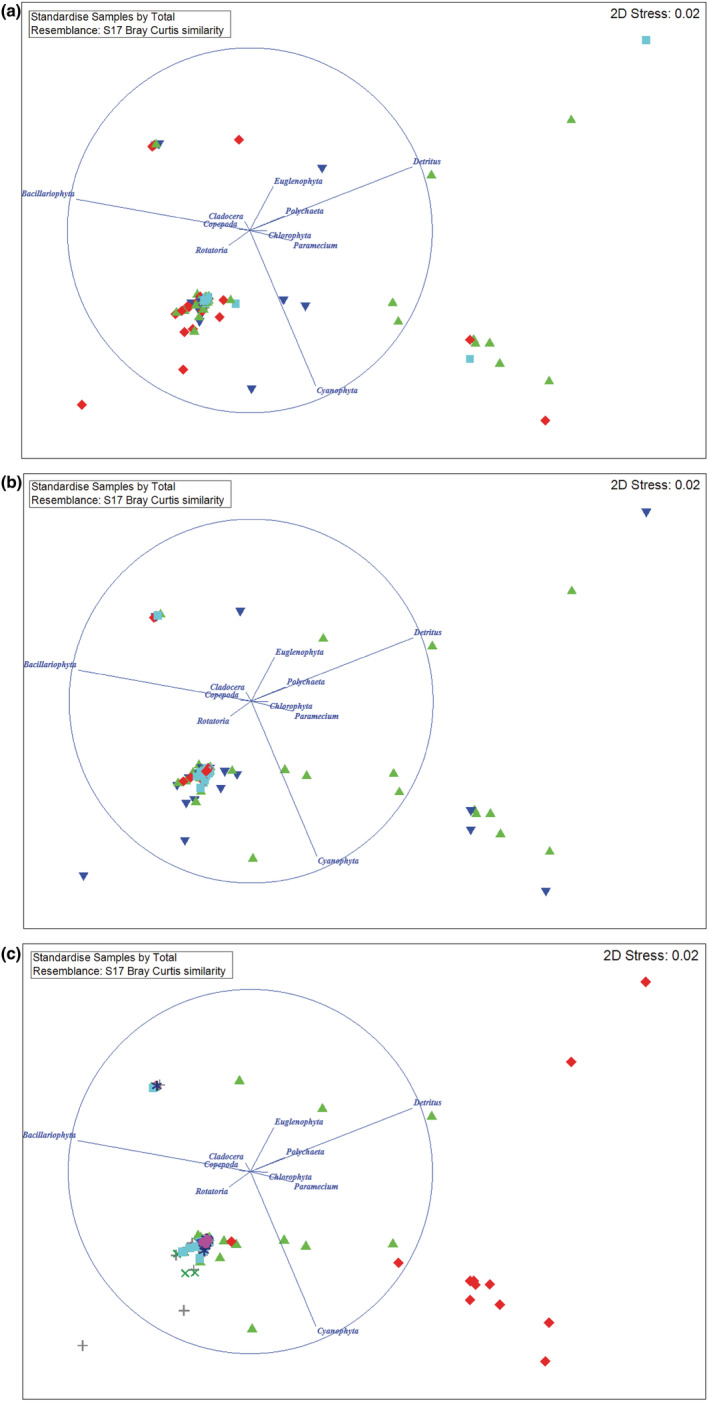
Multidimensional Scaling (MDS) analysis of diet composition of squaretail mullet according to interactions of sex × body size (a) (green triangle: mature male; blue triangle: immature male; turquoise square: mature female; red rhombus: immature female); sex × season (b) (green triangle: male in dry season; blue triangle: female in dry season; turquoise square: male in wet season; red rhombus: female in wet season); and season × site (c) (green triangle: dry season in Tra Vinh; blue triangle: wet season in Tra Vinh; turquoise square: wet season in Ben Tre; red rhombus: dry season in Ben tre; purple circle: wet season in Bac Lieu; +: dry season in Bac Lieu; ×: dry season in Soc Trang; *: wet season in Soc Trang).

The diet composition exhibited variation according to the interaction sex × body size (*Pseudo‐F* = 4.04, *df* = 1, *p* = .04); sex × season (*Pseudo‐F* = 3.60, *df* = 1, *p* = .03); and season × site (*Pseudo‐F* = 12.81, *df* = 3, *p* = .001). The results of the MDS graphs visualized the influence of the above interactions on the diet composition of mullet; and Bacillariophyta, detritus derived from organic matter, and Cyanophyta were still the three main food (Figure 3a–c, respectively). All 2D stress values in Figure 3 were 0.02, indicating that MDS graphs were significant.

Refer to the interaction between sex and body size on the food spectrum of mullet, in the immature group, Chlorophyta was the only food that differed between males and females (*Z* = −2.09, *df* = 1, *p* < .04), whereas, in the mature group, the male and female consumed the same food except for Bacichloropyta (*Z* = −2.93, *df* = 1, *p* = .003). Considering the interaction between sex and season, detritus (*Z* = −5.43, *df* = 1, *p* < .01), Bacillariophyta (*Z* = −3.15, *df* = 1, *p* = .002), Cyanophyta (*Z* = −5.01, *df* = 1, *p* < .01), Chlorophyta (*Z* = −2.33, *df* = 1, *p* = .02), Euglenophyta (*Z* = −6.42, *df* = 1, *p* < .01), and Polychaeta (*Z* = −2.77, *df* = 1, *p* = .006) were the main cause of variation in diet of male in dry and wet season, whereas, in female, Detritus (*Z* = −4.75, *df* = 1, *p* < .01), Bacillariophyta (*Z* = −2.33, *df* = 1, *p* < .01), Cyanophyta (*Z* = −3.20, *df* = 1, *p* = .001), and Euglenophyta (*Z* = −6.3.8742, *df* = 1, *p* < .01) caused a seasonal variation in their diet composition. In the wet season, the dietaries of this mullet at four sampling sites were not statistically different (Kruskal–Wallis test, all *p* > .05). However, in the dry season, their food composition differs according to the sampling sites, due to the variability of the following food items: detritus (Kruskal–Wallis H, *χ* ^*2*^ = 99.22, *df* = 3, *p* < .01), Bacillariophyta (*χ* ^*2*^ = 78.08, *df* = 3, *p* < .01), Cyanophyta (*χ* ^*2*^ = 86.55, *df* = 1, *p* < .01), Chlorophyta (*χ* ^*2*^ = 17.57, *df* = 3, *p* = .001), and Euglenophyta (*χ* ^*2*^ = 139.17, *df* = 3, *p* < .01). Thus, it could be concluded that the diet of this fish changed by sex at different fish sizes in the dry and wet seasons at each sampling site. Conversely, the diet composition was not dependent on the interaction of sex × site (*Pseudo‐F* = 0.82, *df* = 3, *p* = .32), body size × season (*Pseudo‐F* = 1.51, *df* = 1, *p* = .19), and body size × site (*Pseudo‐F* = 0.78, *df* = 3, *p* = .34).

## 4 DISCUSSION

According to Nikolsky (1963) and Al‐Hussaini (1947), fish was classified as omnivores if RGL was 1–3, carnivores if RGL was lower than 1, and herbivorous if RGL was greater than 3. Indeed, the plant‐based matter took a long time to digest, so the RGL of herbivorous fishes was higher than that of omnivorous and carnivorous fishes (Mahasetha, 2016). In the present study, as the average RGL of squaretail mullet was 1.81 and its food was mostly derived from algae, squaretail mullet was classified as algi‐omnivore. The female and mature fish could require more food to have vital energy for breeding, so their RGL values were significantly high in some cases. Previous studies supported the result, for instance, Wijeyaratne and Costa (1990) revealed that *Liza vaigiensis* was omnivorous grazers with RGL ranging from 2.31 to 3.43. Four mullet species from the Shatt Al‐Arab River, Iraq, *Planliza abu*, *P. subviridis*, *P. klunzingeri*, and *Osteomugil speigleri* were herbivorous (Mohamed & Abood, 2019). The feeding habit exhibited great variability depending on species, habitat, season, and even within the same species due to the spatiotemporal change of food composition (Islam et al., 2009). For example, *Mugil cephalus* from Krishnapatnam, India, was omnivorous (Lavanya et al., 2018) but herbivorous in Bangladesh's coastal water (Islam et al., 2009). Coad (2010) stated that mullets could be herbivorous and/or detritivorous fish based on their prey items.

The high FI value (4.39%) indicated the feeding intensity of squaretail mullet was strong. A similar result was found in *Liza vaigiensis*, a synonymized name of squaretail mullet, its feeding intensity ranged from 2.50 to 4.88% (Hajisamae et al., 2004). In the Shatt Al‐Arab River of Iraq, the high feeding intensity was reported in four mullets *Planliza abu*, *P. subviridis*, *P. klunzingeri*, and *Osteomugil speigleri* (Mohamed & Abood, 2019), and *L. subviridis* from Parangipettai waters, India (Rahman et al., 2016). They fed continuously and never stopped feeding year‐round, although their feeding activities and intensity fluctuated monthly. The equivalence of FI values between the wet and dry seasons despite the higher organic matter content in the wet season (Nedeco, 1993). The FI was also similar at the sampling sites, indicating that mullet was well adapted to the environment in the coastal estuary area. In this study, the FI change was not observed between immature and mature fishes, proving that there was no competition for food between them. The opposite result was found in the research of Mondal et al. (2015): the gray mullet of *M. cephalus* had a higher feeding intensity than smaller ones because of the reasons: large fish needed more energy for reproduction and maintenance of larger bodies; and the wider the mouth, so they eat more vigorously. Sometimes, the feeding intensity also depended on the food availability and abundance.

The diet composition showed Bacillariophyceae followed by detritus and Cyanopyta were the main food constituent of squaretail mullet. Brian (2017) reported *E. vaigiensis* consumed diatom, algae, detritus, polychaetes, mollusks, and crustaceans together with sand and mud. *Liza vaigiensis*, in the study of Hajisamae et al. (2004) from coastal line, Singapore ingested mostly microcrustaceans, especially copepods. It was possible that the diet of the squaretail mullet changed to adapt to different habitats. For example, the number and occurrence of food items in *L. subviridis* from Merbok Estuary, Kedah, Malaysia were diatoms (8.4 and 38.92%), algae (2.31 and 9.85%), desmids, plant materials, zooplankton (1.92 and 18.23%), detritus, sand grains, and fishes (Fatema et al., 2013). Nonetheless, on the southeast coast of India, *L. subviridis* consumed mainly detritus and sand, diatoms, dinoflagellates, algae, zooplankton, foraminiferans, polychaetes, and larval (Rahman et al., 2016). Another example, diatoms, algae, molluscans, decayed organic matter, sand and mud, etc. were the prey items of *M. cephalus* in Andhra Pradesh, India (Lavanya et al., 2018), but plant materials, diatoms, algae, and dinoflagellates were the dominant food item of *M. cephalus* in Elechi Creek, Nigeria (Jamabo & Maduako, 2015).

Detritus was the essential food in the diet composition of squaretail mullet and did not vary with sex, body size, season, and sampling site based on its highest IRI values. A different phenomenon was reported by Jamabo and Maduako (2015) that *M. cephalus* adults fed supplemental annelids, fish parts, insect parts, sand/mud, and organic matter compared with juveniles. In *L. vaigiensis* from Sri Lanka, detritus was found to be the most item for 5–10 cm and 20–25 cm size groups; however, serpulid polychaetes were the most abundant in 10–15 cm and 15–20 cm size group (Wijeyaratne & Costa, 1990).

Sex differences in food selection may be due to random selection or to different energy requirements between males and females, but within the scope of this study, the primary and preferred foods item for both sexes were not different. It was evident that males and females of squaretail mullet tended to feed more actively on Bacillariophyta, detritus, and Cyanophyta than other food items. Similarly, *L. subviridis did not change the propensity to feed between males and females; its favorite foods were diatoms, dinoflagellates, zooplankton, and algae (*Rahman et al., 2016 *). Notably, in the study of* Wijeyaratne and Costa *(* 1990 *), the main food item of L. vaigiensis changed by month, but* serpulid polychaetes were always the essential food item*. Specifically*, detritus was the main food item from April to December, while serpulid polychaetes were dominant from January to March in squaretail mullet *with a total length of 5–10 cm*; whereas, in July and October detritus was the major item and in August, sand particles were the most in size group of 20–25 cm.

## 5 CONCLUSION

The squaretail mullet was algi‐omnivorous, and most of its food items were algae‐based, consisting of Bacillariophyta, detritus derived from organic matter, Cyanophyta, Euglenophyta, Chlorophyta, Paramecium, Copepoda, Rotatoria, Polychaeta, and Cladocera. The diet composition changed concerning sex and season. The feeding intensity of this mullet was relatively high and tended to decrease in the breeding season and increase in the post‐breeding season. The knowledge about the food and feeding habits of *E. vaigiensis* in this study provided systematic information on the nutritional biology that is useful in the future artificial culture of this species.

## AUTHOR CONTRIBUTIONS

**Quang Minh Dinh:** Conceptualization (equal); data curation (equal); funding acquisition (equal); investigation (equal); methodology (equal); project administration (equal); resources (equal); validation (equal); writing – original draft (equal); writing – review and editing (equal). **Ngon Trong Truong:** Investigation (equal); methodology (equal); writing – original draft (equal); writing – review and editing (equal). **Ton Huu Duc Nguyen:** Conceptualization (equal); data curation (equal); investigation (equal); resources (equal); writing – original draft (equal); writing – review and editing (equal). **Tran Thi Huyen Lam:** Conceptualization (equal); investigation (equal); methodology (equal); resources (equal); validation (equal); writing – original draft (equal); writing – review and editing (equal). **Tien Thi Kieu Nguyen:** Investigation (equal); methodology (equal); validation (equal); writing – original draft (equal); writing – review and editing (equal). **Dung Quang Le:** Investigation (equal); validation (equal); writing – original draft (equal); writing – review and editing (equal). **Simon Kumar Das:** Investigation (equal); validation (equal); writing – original draft (equal); writing – review and editing (equal).

## FUNDING INFORMATION

This work was funded by VINGROUP and supported by Vingroup Innovation Foundation (VINIF) under project code VINIF.2020.DA01.

## CONFLICT OF INTEREST

The authors declare that they have no competing interests.

## Data Availability

Please find our raw data at https://doi.org/10.5061/dryad.z08kprrg3.
